# Risk Factors for Cardiovascular Disease Mortality Among 86866 Members of the Thai Cohort Study, 2005-2010

**DOI:** 10.5539/gjhs.v7n1p107

**Published:** 2014-08-15

**Authors:** Jiaying Zhao, Matthew Kelly, Chris Bain, Sam-Ang Seubsman, The Thai Team Cohort Study, Adrian Sleigh

**Affiliations:** 1 National Centre for Epidemiology and Population Health, Research School of Population Health, ANU College of Medicine, Biology and Environment, The Australian National University, Acton, Canberra, Australia; 2 Population Health Department, QIMR Berghofer Medical Research Institute, Brisbane, Queensland, Australia; 3 School of Human Ecology, Sukhothai Thammathirat Open University, Nonthaburi, Thailand

**Keywords:** cardiovascular diseases, mortality, Thai Cohort study, cardiovascular risk factors, smoking

## Abstract

Thailand is experiencing a development-associated health-risk transition with increasing prominence of chronic diseases. We aim to determine the risk factors for cardiovascular disease (CVD) deaths in Thailand. We conducted longitudinal analyses of deaths in the nationwide Thai Cohort Study from 2005 to 2010 (n=86866) using national vital registration data. Multivariate logistic regression models were used to calculate mutually adjusted estimates of association between dying from CVD and various risk factors measured at baseline in 2005. For three important risks, population attributable fractions were calculated. There were 78 CVD deaths. The probability of dying from CVD for males was 0.15% and for females was 0.04%. Multivariate modelling showed that current smoking (OR=4.01, CI=2.02-7.93), hypertension (OR=1.91, CI=0.95-3.85), and diabetes (OR=2.51, CI=1.01-6.25) are major risk factors of CVD deaths. For males, 54% of CVD deaths can be attributed to smoking. Females are protected by very low rates of smoking. Ischaemic heart disease (OR=6.85, CI=2.47-19.01) is also a strong predictor of CVD deaths. As CVD is a top cause of death, reducing CVD mortality by controlling smoking, hypertension, and hyperglycaemia will substantially improve life expectancy in Thailand today. The low smoking rates among females need to be actively maintained and confer great benefit.

## 1. Introduction

In Thailand, in response to better nutrition, sanitation and health services over the last 20-40 years, deaths due to infectious disease, maternal mortality and child mortality have fallen (Sleigh, Seubsman, & Bain, 2008). Now, with changing lifestyles, diet and physical activity chronic diseases are rising. National vital statistics, corrected by verbal autopsy, now show cardiovascular disease (CVD) has become a leading cause of death for both men and women (Porapakkham et al., 2005; Kosulwat, 2002). For planning policy responses to such a profound health transition it is best to use national data on relevant risk patterns and associated local cause-specific mortality (Carmichael, 2011). Indeed, national level public health policy and practice are most effective when they respond to local data (Mahapatra et al., 2007). For example, trends in the developed world reveal continued problems with high rates of smoking among females, but in Thailand the female smoking rate remains negligible; evidently, local perspective needs to drive local responses to smoking.

There is limited published information on risk factors for CVD mortality in Thailand. However, two geographically restricted cohort studies (Electricity Generating Authority in Bangkok and the Khon Kaen Cohort Study) have reported that hypertension, diabetes, smoking, cholesterol and male sex are associated with CVD death (Sritara et al., 2003; Kamsa-Ard et al., 2013). National surveys also indicate that some internationally established risk factors for CVD (e.g. overweight and diabetes) have increased significantly in Thailand (Deerochanawong et al., 2013; Ministry of Public Health, 2011).

Since 2005, we have studied a cohort made up of over 85,000 Thai adults residing nationwide, the Thai Cohort Study (TCS). There were 78 CVD deaths in the cohort over the period 2005-2010, enough to provide an early picture of local risks and trends for this mortality category of emerging importance in transitional Thailand. The risks assessed include social, occupational, geographic, economic, environmental, dietary, psychological and behavioural factors, as well as physical activity and medical history. Here we present the risk metrics for those TCS members who died from CVD compared to those who survived over the period from 2005 to 2010. This information provides the detailed nationwide risk-mortality data needed to plan a response. It is the first report on CVD death from the TCS.

## 2. Methods

### 2.1 Baseline Information

We use exposure data (see below) from our Thai Cohort Study (TCS) baseline in 2005. TCS is a large longitudinal study (n=86,866) of the health consequences of socioeconomic development in a country experiencing a rapid transition to chronic diseases (Sleigh, Seubsman & Bain, 2008; Seubsman et al., 2011; Seubsman et al., 2012). Cohort participants resided nationwide and were initially aged 18-87 years with most in the 25-35 year age range. All were distance-learning students enrolled at Sukhothai Thammathirat Open University (STOU) and they provided detailed health-risk information by mailed questionnaire, a reliable technique with such well-educated informants used to mail based information exchange. Cohort members are employed in their local communities and most are of modest means, the constraints that led them to Open University. They represent well the STOU student body and the Thai population for field of study, ethnicity, religion, wealth and geographic residence (Seubsman et al., 2012).

Risk factor (‘exposure’) information gathered included sex, birth year, family history, income, civil status, health service access, the quality of health insurance coverage, cardiometabolic disease (doctor diagnosis of ischemic heart disease, hypertension, hyperlipidaemia, or diabetes), smoking, drinking, physical activity, emotional health problems, height and weight. In other studies we have re-measured self-reported height, weight, diabetes and hypertension all of which were of adequate accuracy for epidemiologic validity (Thawornchaisit et al., 2013; Lim, Seubsman, & Sleigh, 2009).

Reported monthly personal income in Thai Baht (1 US dollar = 35 Baht) was analysed in 4 categories (‘<=3000’, ‘3001−20000’, or ‘20001 and above’). We classified civil status as living with partner or not. Health service access was measured by a question that “in the past 12 months, have you personally used any of the following health services”; answers were “only use public services”, “used private services (at least once)”, and “others–neither public nor private”. We recorded health insurance coverage: “30 Baht scheme” (universal coverage available to all Thais since 2001, initially with a small co-payment of 30 baht or $US0.86), “civil servant benefit scheme”, and “others”. The family history variable, recorded in 2005, noted if mother or father had died from CVD (high blood pressure, heart disease, or stroke).

Health behaviours reported include smoking, alcohol consumption, and physical activities. Smoking was classified as ‘never’, ‘ex-smoker’, or ‘current smoker’. Alcohol consumption was classified as ‘occasional social drinker’, ‘never’, ‘current regular drinker’ or ‘now stopped’. Information on exercise-related weekly physical activity (PA) was obtained by asking: “during a typical week (7-day period) how many times on average do you do each of these physical activities”: ‘walking continuously for at least 10 minutes’; ‘mild exercise for more than 20 minutes’; ‘moderate physical activities for more than 20 minutes’; ‘vigorous physical actives for more than 20 minutes’. Metabolically-adjusted exercise-related PA (number of sessions) was calculated as ‘walking + mild + moderate physical activities + 2*vigorous physical activities’; finally, PA was recoded as ‘less than 7 sessions’ or ‘7 sessions or more’. This derived measure of PA was based on the International Physical Activity Questionnaire and the Active Australia Survey (Banks et al., 2011).

Emotional health problems were measured by the seventh question of the Short Form 8 Health Survey^1^: “during the past 4 weeks, how much have you been bothered by emotional problems (such as feeling anxious, depressed or irritable)?” The responses were categorized into not at all/very little/somewhat *versus* quite a lot/extremely. Body size was based on height and weight, evaluated as Body Mass Index (BMI). The BMI categories used Asian standards: underweight (less than 18.5), normal (18.5 to <23), at risk (23 to <25), obese I (25 to <30), and obese II (>=30) (Banks et al., 2011; Okubo et al., 2007).

### 2.2 Mortality Data

Death data come from the national Vital Registration with the Ministry of Interior and Minster of Public Health in Thailand. The 2005 TCS baseline data were linked to the mortality files using the citizen identification number which is unique for every Thai person. From 2005 to 2010, 580 deaths of cohort members were reported to the Ministry of Interior. Cause of death data were added by the Ministry of Public Health. The completeness of registration of adult deaths in Thailand was 86% from 1950-2000 (Mathers el al., 2005), but over the last decade coverage is thought to have improved to 95% (Prasutkul & Vapattanawong, 2006). For the study we report here, the vital statistics office at the Ministry of Public Health investigated cause of death for almost all those whose deaths were ill-defined by investigating hospital records and performing verbal autopsies. After this adjustment, ill-defined deaths were only 8.4% (N=49) of total deaths and there were 78 deaths from CVD.

### 2.3 Statistical Analysis

We used logistic regression to investigate associations between baseline exposures measured in 2005 and risk of dying from cardiovascular disease over the period 2005-2010. Exposures tested include smoking, drinking, physical activity, hypertension, hyperlipidaemia, diabetes, ischaemic heart disease, abnormal weight, and emotional problems. We also tested sex, birth year (five large subgroups – equivalent to 5 age groups), civil status, income, family history of CVD, health service use, and health insurance. We progressively introduced exposure variables into the model if their coefficients or likelihood ratio tests were statistically significant (p<0.05). Incoming variables were also included if they altered the estimates for other variables by changing their odds ratios by 10% or more. The Hosmer-Lemeshow test was used to confirm that the model was adequately fitted. In the final model, correlations between explanatory variables did not show significant multi-collinearity and the mean variance inflation factor value was 1.24. Individuals with missing data or dying from non-cardiovascular causes were excluded from the multivariate model of cardiovascular mortality, leaving 80585 for final analyses.

For the final statistical analyses, we calculate population attributable fractions (PAF) to estimate the proportion of CVD deaths in the population that would be avoided if certain important risk factors were removed. The formula used and sources of data are given with Table 3, along with the PAFs for smoking, hypertension, and diabetes.

**Table 1:** Demographic characteristics of Thai Cohort study members who died of cardiovascular diseases, 2005-2010

	Cardiovascular Deaths n (%)		Other causes of death n (%)		Survived n (%)		Total n (%)	
Sex								
Males	61	(0.15%)	338	(0.86%)	38998	(98.99%)	39397	(100%)
Females	17	(0.04%)	164	(0.35%)	47288	(99.62%)	47469	(100%)

Birth year								
<1960	24	(0.45%)	88	(1.63%)	5273	(97.92%)	5385	(100%)
1960-1969	22	(0.12%)	121	(0.67%)	17894	(99.21%)	18037	(100%)
1970-1974	9	(0.06%)	81	(0.53%)	15184	(99.41%)	15274	(100%)
1975-1979	11	(0.05%)	97	(0.44%)	21901	(99.51%)	22009	(100%)
1980-1986	12	(0.05%)	115	(0.44%)	26034	(99.51%)	26161	(100%)

**Table 2:** Risk factors for cardiovascular mortality, Thai Cohort Study (2005-2010)*

Risk factors	Number of CVD deaths	Number Surviving**	Adjust OR (95%)	
Sex				
Males	61	38998	ref	
Females	17	47288	0.43	0.21-0.87
Birth cohort				
<1960	24	5273	ref	
1960-1969	22	17894	0.42	0.22-0.82
1970-1974	9	15184	0.19	0.08-0.47
1975-1979	11	21901	0.16	0.06-0.38
1980-1986	12	26034	0.17	0.07-0.41
Civil status				
Not living with a partner	34	44,975	ref	
Living with partner	42	38,789	0.52	0.29-0.94
Drinking				
Occasional social drinker	41	50972	ref	
No, never	16	22429	1.82	0.92-3.60
Current regular drinker	4	4116	0.58	0.20-1.67
Used to drink before now stopped	16	7617	1.64	0.86-3.15
Smoking				
Never	31	60612	ref	
Ex-smoker	20	14540	1.55	0.75-3.19
Current Smoker	25	8603	4.01	2.02-7.93
BMI				
Normal	34	46,372	ref	
Underweight	5	12,517	0.86	0.33-2.24
At risk	12	12,864	0.59	0.28-1.26
Obese I	17	11,201	0.96	0.51-1.83
Obese II	6	2,207	1.72	0.68-4.34
Hypertension				
No	62	82379	ref	
Yes	16	3907	1.91	0.95-3.85
Diabetes				
No	69	85419	ref	
Yes	9	867	2.51	1.01-6.25
Ischaemic heart disease				
No	72	85936	ref	
Yes	5	350	6.85	2.47-19.01

* All odds ratios mutually adjusted

** Deaths from other causes excluded.

**Table 3:** Odds ratio (OR), risk factor prevalence and population-attributable risk (PAF)* of major risk factors for CVD deaths in Thai population

Risk Factors	OR		Prevalence of Risk Factors (%)**		PAF (%)	

	Total	Males	Total	Males	Total	Males
Current Smoking	4.0	3.9	20.7	40.4	38.4	53.9
Hypertension	1.9	1.8	21.5	23.0	16.4	14.9
Diabetes	2.5	3.0	7.5	6.6	10.2	11.8

*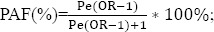 Pe is the prevalence of the risk factor in the Thai population (Webb & Bain 2011);

** Prevalence in National Health Examination Survey, 2009 (Minister of Public Health 2011).

## 3. Results

Of 86866 cohort members analysed, 78 members died from CVD over the 5 years following the 2005 baseline survey (Table 1). The probability of dying from CVD for males (0.15%) was higher than for females (0.04%). The oldest group (born before 1960) had the highest probability of dying from CVD (0.45%) and the youngest birth cohorts (born from 1975 to 1986) had the lowest probability.

The multivariate odds ratios for each explanatory variable in the final model of CVD deaths are shown in Table 2. The estimates were mutually adjusted and the model fit the data well (Hosmer-Lemeshow test, p=0.73). Males had higher mortality odds and risk increased with age. Living with a partner lowered mortality significantly. Compared to never smokers, current smokers were at much higher risk of dying (OR=4.01, CI=2.02-7.93). Ex-smokers also had a higher risk of dying from CVD (OR=1.55, CI=0.75-3.19) but the risk was less than for current smokers. Over the observed time period (5 years) drinking categories were not significantly associated with dying from CVD.

Self-reported ischaemic heart disease was a very strong predictor of CVD death (OR=6.85, CI=2.47-19.01). In addition, people who reported having hypertension (OR=1.91, CI=0.95-3.85) or diabetes (OR=2.51, CI= 1.01-6.25) in 2005 had a greater risk of dying from CVD within five years and this increased risk was statistically significantly for diabetes and nearly significant for hypertension. There was an increased risk (OR=1.72, CI=0.68-4.34) of dying for obese people (BMI>=30), but this association was not significant in our time frame. However, if hypertension and diabetes were excluded in the model, the risk of dying for obese II people would increase to 2.26 (CI=0.92-5.51).

We also tested income, family history of dying from CVD, health services access, quality of health insurance, physical activity, emotional problems, and hyperlipidaemia. No significant relationship between these factors and dying from CVD within the five years was observed.

Table 3 summarises the population effects of smoking, hypertension, and diabetes. For these important risk factors, the table brings together the relative odds of CVD mortality estimated from our Thai Cohort Study, the population prevalence of these risk factors for adults reported by a recent national survey and the estimated population-attributable fractions (PAF%). As there were few (17) female deaths in our cohort we did not calculate the odds ratio or the fractions for females because estimates were too imprecise. We calculated our results for the whole population and then repeated the calculations restricted to males. The estimates for males used odds ratios from a male-only model of CVD deaths that included all the variables shown in Table 2. Odds ratios used for the whole population are listed in Table 2.

Our calculations show that hypertension accounts for 16% and diabetes for 10% of deaths from CVD in the Thai adult population. Current smoking is an even bigger problem as it caused 38% of CVD deaths in the Thai population and 54% of CVD deaths in the male portion of the population. This is indicates the current importance of smoking among Thai males. Also important are the data (not shown) for smoking rates among Thai females which remained very low both in the cohort (1%) and the general population (2%).

## 4. Discussion

Although the deaths in this relatively young nationwide Thai cohort are not yet numerous the associated risk factors cohere well to the pattern reported in developed countries around the world. This implies that CVD in Thailand is going to pose a substantial burden in the next 20-30 years. Our most important cohort-based observation was that among Thai males 54% of future CVD deaths could be avoided by prevention of smoking uptake. Also noteworthy is the related benefit conferred on Thai females by the very low smoking rates in that half of the population; maintenance of such low rates should be a fundamental public health goal for Thailand.

We also demonstrated that hypertension and diabetes are major current risk factors for deaths from CVD for Thais. In the whole population, PAF analyses reveals that 16% of CVD deaths are due to hypertension, and 10% are due to diabetes. As the prevalence of diabetes is rapidly increasing in Thailand (Deerochanawong et al., 2013), it can be expected that this disease will play a bigger role for CVD deaths in the future. Self-reported ischaemic heart disease is also a strong predictor of CVD deaths. BMI >=30 was also associated with dying from CVD, but this effect was not statistically significant in the relatively short time period monitored. Living with a partner halves the risk of dying from CVD. Social drinking was protective with a non-significant decrease in CVD mortality compared with those who never drank or those who stopped drinking.

Similar effects on CVD for smoking have been reported in other Asian populations (Asia Pacific Cohort Studies Collaboration, 2005). For example, in China, smoking is one of the most important contributors to the development of coronary heart disease (Zhang, Lu, & Liu, 2008). Our analyses also suggested that risk of dying from CVD attenuated when people stopped smoking. This is consistent with previous research showing that there is a substantial decrease in CVD mortality for former smokers compared with continuing smokers (Ockene & Miller, 1997; Critchley & Capewell, 2003). So prevention of smoking uptake is the most powerful intervention but cessation of smoking is also very effective for preventing CVD deaths.

In previous research, mostly focused on European populations, obesity has been associated with diabetes, hypertension, stroke, and ischaemic heart disease (Haslam & James, 2005). Recently, Zheng et al. (2011) have pooled the results for 120,700 deaths in 19 Asian cohort studies and showed that deaths from CVD increased when BMI exceeded 27.5. One other study in Thailand reported a weak increase in risk of all-cause mortality associated with BMI >= 35 among older men (Vapattanawong et al., 2010). In our study, the increased risk of deaths was unequivocal once BMI reached 30. But we note our cohort is still young and there were relatively few deaths observed over the relative short 5 year period. Also, much of the obesity effect on CVD mortality may operate through a causal pathway that includes diabetes and hypertension. When diabetes and hypertension were excluded in our model, the obesity-related risk of CVD death increased, approaching statistical significance.

The relationship between marital status and mortality has been repeatedly demonstrated in a large number of countries (Hu & Goldman, 1990). Married individuals have higher emotional and social support and lower stress (Lipowicz & Lopuszanska, 2005). Although psychological problems were controlled in our analyses, the protective effect of living with a partner remained significant. It is possible that social support may reduce CVD mortality not only through psychological factors but also through health care as well as through first-aid of CVD.

This study is one of the first attempts to link a large longitudinal study on multiple health risk factors with official CVD mortality data in South East Asia. National cause-of-death data in Thailand ten years ago had a high proportion of ill-defined deaths and inaccurate information (Porapakkham et al., 2005). However, in our study, after investigations by Ministry of Public Health, ill-defined deaths were reduced to 8.4% of total deaths and this decreased a major source of error. However, there may have been some other errors when measuring exposure variables in 2005.

In addition, our logistic regression may suffer from the problem of rare events data as there are only 78 CVD deaths over the five years (King & Zeng, 2001). To respond to this possibility, we re-tested the risk factor estimates of our models using both Exact Logistics Regression and Rare Events Logistic Regression, and the results varied by less than 10% to those we obtained from standard logistics regression. Furthermore we repeated the model underlying Table 2 using survival analysis and Poisson regression and the resulting Hazard Ratios and Incident Risk Ratios for each variable were virtually identical to the Logistic Regression estimates.

## 5. Conclusion

Cardiovascular disease is the top cause of death in Thailand, accounting for 27% of the mortality burden (Institute for Health Metrics and Evaluation, 2013). Mortality from CVD due to smoking, hypertension and diabetes is playing an important role in overall mortality with high population attributable fractions (38%─smoking, 16%─hypertension; 10%─diabetes). Given the high population prevalence of these three key risk factors, reducing CVD mortality by controlling smoking, hypertension, and hyperglycaemia will substantially improve life expectancy in Thailand today. Also important is the need for Thais to be conscious of preserving their culturally conferred population health advantage and ensure that the female half of population continues to avoid smoking.
